# Active Surveillance in Patients with Extra-abdominal Desmoid-Type Fibromatosis: A Pooled Analysis of Three Prospective Observational Studies

**DOI:** 10.1158/1078-0432.CCR-24-2340

**Published:** 2024-12-02

**Authors:** Chiara Colombo, Stefanie Hakkesteegt, Axel Le Cesne, Francesco Barretta, Jean-Yves Blay, Dirk J. Grünhagen, Nicolas Penel, Laurent Lam, Marco Fiore, Elena Palassini, Giovanni Grignani, Francesco Tolomeo, Paola Collini, Alessandra Merlini, Federica Perrone, Silvia Stacchiotti, Cornelis Verhoef, for the Dutch Grafiti Group, Sylvie Bonvalot, for the French Sarcoma Group, Alessandro Gronchi

**Affiliations:** 1 Sarcoma Service–Department of Surgery, Fondazione IRCCS Istituto Nazionale dei Tumori, Milano, Italy.; 2 Department of Surgical Oncology, Erasmus MC Cancer Institute, University Medical Center Rotterdam, Rotterdam, the Netherlands.; 3 Department of Medical Oncology, Institut Gustave Roussy, Villejuif, France.; 4 Department of Biostatistics for Clinical Research, Fondazione IRCCS Istituto Nazionale dei Tumori, Milano, Italy.; 5 Department of Medical Oncology, Centre Leon Berard, Lyon, France.; 6 Department of Medical Oncology, Centre Oscar Lambret, Lille, France.; 7 Department of Biostatistics, Institut Gustave Roussy, Villejuif, France.; 8 Cancer Medicine Department, Fondazione IRCCS Istituto Nazionale dei Tumori, Milano, Italy.; 9 Department of Oncology, AOU Città della Salute e della Scienza di Torino, Torino, Italy.; 10 Department of Oncology, University of Turin, Orbassano, Italy.; 11 Soft Tissue Tumor Pathology Unit, Department of Advanced Diagnostics, Fondazione IRCCS Istituto Nazionale dei Tumori, Milano, Italy.; 12 Department of Surgical Oncology, Institut Curie, Paris, France.

## Abstract

**Purpose:**

Three prospective observational studies (Italy, the Netherlands, and France) on active surveillance (AS) in patients with extra-abdominal desmoid-type fibromatosis support AS as a first-line approach. Identifying prognostic factors for the failure of AS will help determine the strategy. The aim of this study was to investigate the prognostic impact of clinical and molecular variables in a larger series.

**Experimental Design:**

Data available as of January 31, 2024, from the three studies, in which patients were followed for ≥3 years, were pooled. Patients ≥18 years of age, with primary sporadic desmoid-type fibromatosis, and with *CTNNB1* mutations available were eligible. The primary study endpoint was treatment-free survival (TFS). Secondary endpoints included the incidence of RECIST progression, spontaneous RECIST regression, and regression post-RECIST progression.

**Results:**

Patients (*n* = 282) with a median follow-up of 53 months (IQR, 39–63) were included. The 3- and 5-year TFS rates were 67% and 66%, respectively; the 3- and 5-year crude cumulative incidences were 33% and 34% for RECIST progression, 26% and 34% for RECIST regression, and 33% and 38% for regression post-RECIST progression, respectively. In multivariable analysis, larger tumor size, mutation type, and tumor locations were associated with lower TFS. The specific mutation (S45F), larger tumor size, and extremity and trunk locations were all associated with a lower probability of spontaneous RECIST regression.

**Conclusions:**

This study confirms that spontaneous regression occurs in a significant proportion of patients and that two-thirds are treatment free at 5 years. Initial tumor size, *CTNNB1* mutation, and location should be factored into the initial decision-making process.

**Translational Relevance**

Desmoid-type fibromatosis is a rare mesenchymal disease that affects predominantly young adults with an unpredictable clinical behavior characterized by spontaneous growth arrest and prolonged stabilization without any specific treatment. Active surveillance represents the initial strategy for the majority of patients. However, approximately 30% need active treatments, but prognostic factors are not yet available. In this pooled analysis from three prospective observational studies on patients with extra-abdominal desmoid-type fibromatosis, we confirm that spontaneous regression occurs in a significant proportion of patients and that two-thirds are treatment free at 5 years. Moreover, we found that the specific mutation (S45F), larger tumor size, and extremity and trunk locations were all associated with a lower probability of spontaneous RECIST regression. Initial tumor size, *CTNNB1* mutation, and location should be factored into the initial decision-making process.

## Introduction

Desmoid-type fibromatosis (DTF) is a rare, nonmetastasizing, intermediate-grade soft-tissue tumor. DTF tumors can arise in any part of the body, with young adults being primarily affected (1). The majority of DTF tumors are sporadic and characterized by mutations in exon 3 of the β-catenin (*CTNNB1*) gene, including T41A, S45F, and S45P, in most patients (2). The biological behavior of DTF is unpredictable, with phases of initial progression, long-term disease stabilization, or spontaneous regression without any treatment. Active surveillance (AS) is currently recommended as the first-line approach for the management of DTF (3). In recent years, three European studies [France (NCT 01801176), Italy (NCT 02547831), and the Netherlands (Netherlands Trial Register NTR4714)] provided the first prospective confirmation that the majority of patients with DTF eventually develop stable or regressive disease, even after initial progression (4–6). One-third of the patients in these studies needed active treatment (AT) after an initial AS approach, and tumor size, tumor location, and *CTNNB1* mutation status were identified as potential predictors for the start of AT. Based on the individual studies, the potential correlation between *CTNNB1* mutation status and tumor behavior was not fully understood. The analyses of clinicopathologic factors associated with DTF tumor behavior and the start of AT were limited by the relatively small number of patients in the individual study cohorts. The aim of the current study was to strengthen the evidence from the previous prospective studies and to further test prognostic factors for the failure of AS and tumor behavior by combining the results of the Italian, Dutch, and French prospective studies. The ability to predict tumor behavior and determine which patients will need AT will allow for an individualized approach in terms of treatment and follow-up (FU) schedules for patients with extra-abdominal DTF.

## Materials and Methods

### Study design and population

Individual data from patients with DTF who were followed during AS in the Italian, Dutch, and French prospective studies (NCT02547831, inclusion 2013–2018; NTR4714, inclusion 2014–2018; and NCT 01801176, inclusion 2012–2015) were pooled. The study protocols were approved by institutional review boards according to the applicable laws at the participating centers. Written informed consent was obtained from all patients. The study was conducted in accordance with the provisions of the Declaration of Helsinki. As there were minimal differences between the inclusion and exclusion criteria of the original studies, the following inclusion criteria were applied to ensure a uniform study population for the combined analyses: ages ≥18 years, primary extra-abdominal sporadic DTF, and a known *CTNNB1* mutation status. The study design and procedures of the original studies have been published previously (4–6). Briefly, all patients observed at the participating institutions in the three countries were initially placed on AS and included in the original studies. FU was based on clinical and radiological evaluation according to the previously published data (4–6). The protocols did not require a specific time interval between the data of the biopsy and the time of the first baseline radiological evaluation. However, for all patients, the initial size was registered from the first imaging that was available before biopsy and then compared with the first imaging after enrollment. In general, the time interval was 3 to 4 months. Tumor behavior of DTF was evaluated according to RECIST version 1.1 (7). The decision to start AT was made individually by both the physician and patient and was discussed in a multidisciplinary meeting. Reasons for reevaluating the current AS management strategy were tumor growth or progressive symptoms according to international guidelines. Therapy administered on an individualized basis included surgery, radiotherapy, and systemic therapies, including hormonal therapy (e.g., tamoxifen or toremifene), low-dose chemotherapy (e.g., methotrexate, cyclophosphamide, vinorelbine/vinblastine, or a combination of these types), COX inhibitors (e.g., celecoxib), targeted therapies (imatinib), and combination therapy (hormonal therapy plus COX inhibitor). When AT was started, radiological and/or symptomatic progression was registered. The end of FU was marked by the start of AT or the last registered contact between the physician and the patient. All data available at the time of the analyses (January 2024) were used for the pooled analyses.

### Statistical methods

The primary study endpoint was treatment-free survival (TFS). Secondary endpoints were the incidence of RECIST disease progression (PD, ≥20% spontaneous increase in the longest diameter), RECIST spontaneous regression (RR, ≥30% spontaneous decrease in the longest diameter), spontaneous regression of any entity post–RECIST progression (RpPD), first spontaneous regression (FR, spontaneous reduction of any entity of the longest diameter), RECIST spontaneous regression as any event (RRae), and progression-free survival (PFS).

Patient and disease characteristics and treatments are summarized using descriptive statistics.

TFS was defined as the time elapsing from the initial biopsy to any AT. PFS was defined as the time elapsing from the initial biopsy to the first RECIST progression. Time was censored at the latest FU for patients still under AS or not relapsed. The TFS and PFS curves were estimated using the Kaplan–Meier method and compared using the log-rank test. RECIST PD was defined as the time elapsing from the initial biopsy to the first RECIST progression. RpPD was defined as the time elapsing from the first RECIST PD to the first spontaneous regression of any entity. FR was defined as the time elapsing from the initial biopsy to the first spontaneous regression of any entity in patients who did not experience RECIST PD. RR was defined as the time elapsing from the initial biopsy to RECIST spontaneous regression as the first event. RRae was defined as the time elapsing from the initial biopsy to the RECIST spontaneous regression either as the first event or as the secondary event after progression. Crude cumulative incidence (CCI) curves for PD, RpPD, FR, RR, and RRae were estimated in a competing risk setting using cumulative incidence estimates and compared using the Gray test. For PD, FR, and RRae, the competing event was AT. For RpPD, the competing event was AT after initial RECIST progression. For RR, the competing events were AT and RECIST progression.

Univariable and multivariable Cox (for TFS) and Fine and Gray (for PD, RpPD, FR, RR, and RRae) models were run to assess the influence of sex (male or female), age (continuous), initial tumor size (continuous and categorized as <50 or ≥50), *CTNNB1* mutation type (T41A, S45F, S45P, “other,” or wild-type for both *CTNNB1* and *APC*), and tumor location (extremities, abdominal wall, head/neck, or trunk) on these outcomes. Model results are reported as HR for the Cox models and subdistribution HR (sHR) for the Fine and Gray models, with 95% confidence intervals (CI) and Wald or Gray test *P* values. Age and size (as continuous covariates) were modeled using three-knot restricted cubic splines (8).

The median FU times were estimated by the reverse Kaplan–Meier method from the TFS data (9).

Statistical analyses were carried out using SAS (version 9.4, SAS Institute, Cary, NC) and R software (version 4.1.2, R Foundation for Statistical Computing).

### Data availability

Deidentified individual patient data are available from the corresponding author upon reasonable request.

## Results

Of the 313 patients included in the individual studies, 282 were eligible for the current study and were included in the pooled analyses (Supplementary Figs. S1 and S2). Baseline characteristics are detailed in Table 1 and Supplementary Table S1. The median FU was 52 months (IQR, 38–64). No patient died during the study period.

**Table 1.** Baseline characteristics of 282 included patients with DTF.

Characteristic	*n* (%)
Age at diagnosis (years)	
Median (first and third quartile)	38 (31–47)
Sex	
Male	46 (16.3)
Female	236 (83.7)
Tumor localization	
Abdominal wall	134 (47.5)
Head and neck	11 (3.9)
Extremities	61 (21.6)
Trunk	76 (27.0)
Tumor size (mm)	
Median (first and third quartile)	50 (35–74)
<50	129 (45.7)
≥50	153 (54.3)
*CTNNB1* mutation status	
T41A	156 (55.3)
S45F	44 (15.6)
S45P	30 (10.6)
Other	15 (5.3)
WT	37 (13.1)

Abbreviation: WT, wild-type.

### TFS

**Figure 1.**
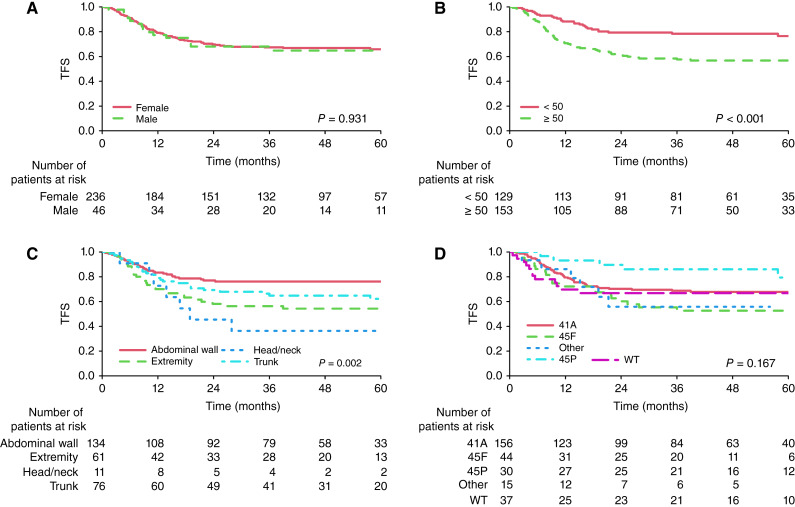
Kaplan–Meier curves for TFS according to sex (**A**), initial tumor size in mm (**B**), tumor locations (**C**), and *CTNNB1* mutation types (**D**). WT, wild-type.

The 3- and 5-year TFS rates (95% CI) were 67% (62%–73%) and 66% (60%–72%), respectively (Supplementary Fig. S2A). In univariable analysis, larger tumor size (*P* < 0.001) and head/neck and extremity tumor locations (*P* = 0.002) were associated with a worse TFS (Fig. 1A–D). In multivariate analysis, larger tumor size (HR, 70 vs. 20: 3.75; 95% CI, 1.82–7.76; *P* < 0.001), mutation type (HR, other vs. S45P: 3.93; 95% CI, 1.24–12.47; S45F vs. S45P: 1.63; 95% CI, 0.64–4.18), and tumor locations (HR, head and neck vs. abdominal wall: 2.92; 95% CI, 1.20–7.10; extremities vs. abdominal wall: 1.76; 95% CI, 1.00–3.12) were associated with lower TFS (Table 2).

**Table 2.** Results of the multivariable Cox and Fine and Gray models for TFS, PD, RpPD, FR, and RR.

	TFS			PD			RpPD			FR			RR		
	HR	(95% CI)	*P*	sHR	(95% CI)	*P*	sHR	(95% CI)	*P*	sHR	(95% CI)	*P*	sHR	(95% CI)	*P*
Sex			0.555			0.480			0.496			0.420			0.946
Male vs. female	0.84	(0.47–1.50)		1.20	(0.72–2.02)		1.41	(0.52–3.85)		0.79	(0.45–1.40)		1.02	(0.53–1.98)	
Age at diagnosis^a^ (years)			0.819			0.579			0.657			0.051			0.314
45 vs. 20	0.79	(0.37–1.68)		0.62	(0.26–1.51)		2.18	(0.41–11.55)		2.18	(0.98–4.82)		1.73	(0.73–4.10)	
70 vs. 45	1.12	(0.60–2.09)		1.15	(0.63–2.11)		0.80	(0.25–2.55)		0.52	(0.28–0.97)		0.52	(0.20–1.39)	
Initial tumor size^a^ (mm)			<0.001			0.423			0.052			0.051			0.003
70 vs. 20	3.75	(1.82–7.76)		0.79	(0.44–1.40)		0.71^b^	(0.23–2.16)^b^		0.62	(0.37–1.04)		0.42	(0.23–0.76)	
100 vs. 70	1.19	(1.07–1.33)		1.08	(0.94–1.23)		0.17^b^	(0.03–0.79)^b^		0.75	(0.46–1.23)		0.73	(0.47–1.11)	
*CTNNB1* mutation types			0.188			0.923			0.248			0.710			0.517
S41A vs. S45P	1.59	(0.68–3.74)		1.09	(0.57–2.07)		0.63	(0.29–1.39)		1.35	(0.83–2.19)		0.86	(0.49–1.51)	
S45F vs. S45P	1.63	(0.64–4.18)		0.95	(0.43–2.09)		0.24	(0.02–2.46)		1.15	(0.56–2.38)		0.49	(0.19–1.25)	
Other vs. S45P	3.93	(1.24–12.47)		1.47	(0.55–3.95)		0.17	(0.03–0.94)		0.94	(0.38–2.35)		0.57	(0.21–1.58)	
Wild type vs. S45P	2.06	(0.75–5.68)		0.96	(0.43–2.16)		1.00	(0.30–3.38)		1.10	(0.53–2.31)		0.71	(0.31–1.50)	
Tumor location			0.070			0.161			0.295			0.081			0.012
Extremities vs. abdominal wall	1.76	(1.00–3.12)		1.08	(0.61–1.92)		0.36	(0.08–1.51)		0.53	(0.31–0.92)		0.59	(0.13–0.65)	
Head and neck vs. abdominal wall	2.92	(1.20–7.10)		2.21	(1.03–4.74)		0.23	(0.02–2.35)		1.31	(0.48–3.58)		0.44	(0.11–1.75)	
Trunk vs. abdominal wall	1.54	(0.86–2.77)		0.99	(0.55–1.77)		0.96	(0.42–2.17)		0.76	(0.45–1.26)		0.57	(0.33–1.00)	

a Modeled as restricted cubic spline.

b The values compared for this endpoint were 50 vs. 20 and 100 vs. 50.

### RECIST PD

**Figure 2.**
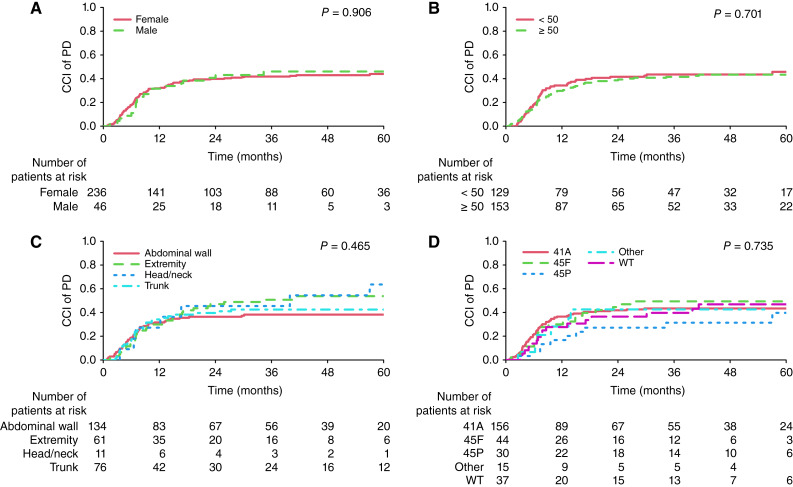
CCI curves for RECIST progression according to sex (**A**), initial tumor size in mm (**B**), tumor locations (**C**), and *CTNNB1* mutation types (**D**). WT, wild-type.

The 3- and 5-year overall CCI (95% CI) of RECIST PD were 43% (37%–49%) and 45% (39%–51%), respectively (Supplementary Fig. S2B). In both univariable and multivariable analyses, none of the assessed covariates were associated with CCI of RECIST PD (Fig. 2A–D; Table 2).

### RR

**Figure 3.**
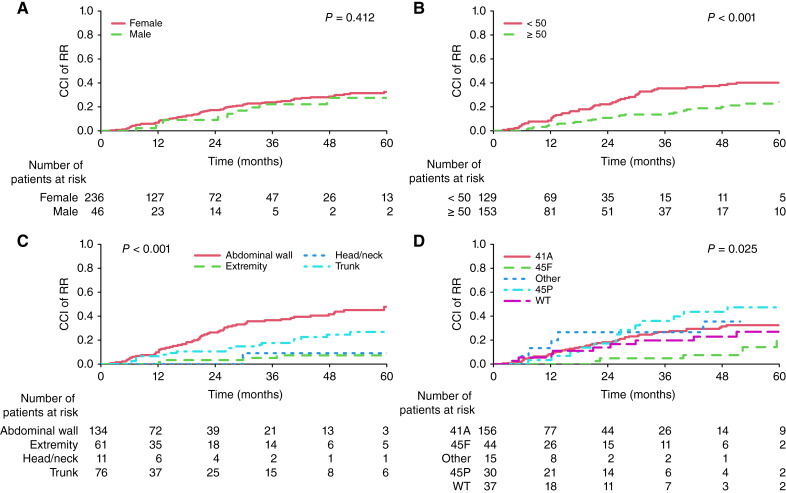
CCI curves for RR according to sex (**A**), initial tumor size in mm (**B**), tumor locations (**C**), and *CTNNB1* mutation types (**D**). WT, wild-type.

Regression according to RECIST as the first event was observed in 82 (29.1%) patients. The 3- and 5-year overall CCI (95% CI) of RR were 24% (19%–29%) and 32% (26%–38%), respectively (Supplementary Fig. S2C). In univariable analysis, age (*P* = 0.010), tumor size (*P* = 0.001), mutation type (*P* = 0.025), and location (*P* < 0.001) were associated with CCI of RR (Fig. 3A–D). In multivariable analysis, larger tumor size (sHR, 70 vs. 20: 0.46; 95% CI, 0.25–0.84; *P* = 0.014) and extremity locations (sHR extremity vs. abdominal wall: 0.22; 95% CI, 0.09–0.57; *P* = 0.010) maintain their statistical significance (Table 2).

### RpPD

A decrease in tumor size after initial RECIST progression was observed in 40 of 123 (32.5%) progressive patients. The 3- and 5-year overall CCI (95% CI) of RpPD were 32% (24%–42%) and 36% (28%–47%), respectively (Supplementary Fig. S2D). In univariable analysis, larger tumor size (*P* = 0.023) was significantly associated with lower CCI of RpPD (Supplementary Fig. S3A–S3D). In multivariable analysis, only tumor size larger than 50 mm (sHR, 100 vs. 50: 0.14; 95% CI, 0.02–0.82; *P* = 0.022) shows a significant association with lower CCI of RpPD (Table 2).

### FR

The 3- and 5-year overall CCI (95% CI) of FR were 71% (64%–78%) and 78% (71%–86%), respectively (Supplementary Fig. S2E). In univariable analysis, age (sHR, 45 vs. 20: 1.87; 95% CI, 0.92–3.82; sHR, 70 vs. 45: 0.36; 95% CI, 0.19–0.69; *P* = 0.006), tumor size (*P* = 0.006), and location (*P* = 0.020) were significantly associated with CCI of FR (Supplementary Fig. S4A–S4D). In multivariable analysis, older age (sHR, 45 vs. 20: 1.35; 95% CI, 0.63–2.87; sHR, 70 vs. 45: 0.49; 95% CI, 0.25–0.95) and larger size (sHR, 100 vs. 20: 0.41; 95% CI, 0.21–0.79, comparison not in table) remained significantly associated with lower CCI of FR. In addition, the extremity location (sHR extremity vs. abdominal wall: 0.56; 95% CI, 0.31–1.01) level was singularly associated with lower CCI of FR (Table 2).

### RRae

The 3- and 5-year overall CCI of RRae were 32% (27%–38%) and 44% (38%–51%), respectively (Supplementary Fig. S2F). In univariable analysis, age (HR, 45 vs. 20: 2.12; 95% CI, 1.10–4.09; HR, 70 vs. 45: 0.30; 95% CI, 0.12–0.70; *P* = 0.011), tumor size (*P* < 0.001), tumor location (*P* < 0.001), and mutation types (*P* = 0.007) were associated with CCI of RRae (Supplementary Fig. S5A–S5D). In multivariable analysis, extremity and trunk locations (HR, 0.24; 95% CI, 0.11–0.51 and HR, 0.55; 95% CI, 0.34–0.89, respectively; *P* = 0.001) and larger tumor size (HR, 70 vs. 20: 0.43; 95% CI, 0.26–0.70; HR, 100 vs. 70: 0.58; 95% CI, 0.36–0.94; *P* < 0.001) remained significant (protective from RRae). The S45F mutation (HR, S45F vs. S45P: 0.42; 95% CI, 0.18–0.97) was significantly associated with lower CCI of RRae.

### PFS

The 3- and 5-year PFS rates (95% CI) were 54% (48%–60%) and 51% (45%–58%), respectively (Supplementary Fig. S2G). In both univariable and multivariable analyses, none of the assessed covariates were associated with PFS (Supplementary Fig. S6A–S6D).

### Treatment strategy during FU

In total, 93 of 282 (33.0%) patients received AT after initial AS, of which 45 (48.4%) patients received AT after RECIST progression, and 48 (54.6%) patients received AT after symptomatic progression alone. The treatment strategy is summarized in Supplementary Fig. S7.

## Discussion

The results of this combined analysis bolster the evidence that AS can be safely recommended for patients with extra-abdominal DTF. The majority of patients with DTF undergoing AS experience stabilization or spontaneous regression of the disease even after initial progression. Initial tumor size and tumor location were confirmed as prognostic factors for tumor behavior and the need for AT. The *CTNNB1* mutation status despite not being uniformly statistically significant showed a consistent trend toward a worse behavior for S45F and rarer *CTNNB1* mutation types. Around 50% of all patients experienced spontaneous regression either as the first event or following progression, and initial tumor size, tumor location, and *CTNNB1* mutation status were consistently associated with the probability of spontaneous tumor regression. This event was also observed in the placebo arms of the two randomized studies on sorafenib (10) and, more recently, on nirogacestat (11), strengthening the importance of AS in selecting patients who truly need an AT.

### Strength of our study

Previous studies of prognostic factors for tumor behavior and treatment outcomes in DTF have shown conflicting results, most likely because they were all small retrospective studies with heterogeneous patient cohorts, different treatment regimens, and varied outcome measures (12, 13). Our combined prospective study in a uniquely large population of patients with primary DTF, who had no prior AT, allows for the assessment of true prognostic factors and the natural behavior of DTF more accurately than all previous retrospective studies (12–15) and also the placebo arms of the two randomized studies mentioned previously (10, 11). In those studies, patients included had either primary or recurrent disease and could have received various locoregional or systemic treatments before study entry, making it difficult to properly assess the natural history of the disease and the influence of clinical and molecular variables.

### Role of mutation

Specific *CTNNB1* mutations were associated with the failure of AS in the current study, with a higher need for AT in patients with S45F or rarer *CTNNB1* mutation types (classified as “other”). Previous studies also observed that S45F-mutated tumors seem to have a more aggressive character, given their higher risk of recurrence in patients who underwent surgery (16–19). However, this more aggressive behavior does not seem to be due to progression, as no association between *CTNNB1* mutation and RECIST progression was found in our study. A worse pain may have played a role, but we were not able to explore the association between the degree of pain and mutational status, which deserves further studies. Progression according to RECIST represents a limitation in this specific disease, as variations in tumor diameter can occur in the anteroposterior or transverse diameter, not just in the longitudinal one, which is often the longest. The association between the mutation type and probability of RR was observed for the first time in our study, with a lower incidence of regression in patients with S45F mutations. Thus, the extent to which a DTF tumor is able to spontaneously regress could be an indicator of its aggressiveness and could explain the more aggressive nature and worse treatment outcomes for patients harboring an S45F mutation. The underlying mechanism for the differences in the probability of tumor regression between mutation types warrants further investigation. In addition to tumor-related factors, the patient’s immune environment might play a role, as a previous study showed that S45F-mutated DTF tumors have less expression of anti-inflammatory genes than T41-mutated tumors (20).

### Role of size

Larger tumor size at baseline was independently associated with a higher probability of starting AT because of the lower probability of RR. Treating smaller tumors is preferable to treating larger tumors. As the percentage of spontaneous growth arrest and regression is high, the decision to treat a patient should always consider this possibility. Overtreating patients is also a risk, as side effects can occur with any treatment. AS helps identify patients who truly need treatment by showing consistent tumor progression across repeated assessments. If the progression is rapid (though rare, it is possible), one should consider treating the patient sooner rather than later, as also recommended by the recent consensus document (3).

### Role of location

Tumor location, especially in the extremities, was found to be associated with the need for AT and a lower probability of spontaneous regression. It seems plausible that this is because these patients experience more pain and functional problems, leading to impaired health-related quality of life (HRQoL), particularly for extremity sites. Additionally, there may be potential life-threatening sequelae in head/neck locations. Of note, the S45F mutation is more common at these locations (Supplementary Table S1), and this may well be another reason why extremity- and head/neck-located desmoid tumors require treatment more often than desmoid tumors at other locations.

The limitations of the individual studies, including the lack of objectively predefined criteria for symptomatic progression or the initiation of adjuvant therapy, as well as assessment by RECIST, which is largely inadequate in DTF, have been described in detail previously (4–6). Despite the larger number of patients in this combined analysis, the number of patients in some subgroups was still low, such as the number of patients with head/neck tumors and “other” *CTNNB1* mutations. Conclusions for these subgroups should be applied cautiously. In addition to the objective endpoints reported here, HRQoL outcomes are of great importance when evaluating the efficacy of an AS approach in DTF. Although the current study does not report HRQoL outcomes, there is no reason to believe that the HRQoL outcomes of the combined analysis would differ from those of the Dutch prospective study, which demonstrated that an initial AS approach does not impair the HRQoL of patients with DTF who continue AS (21).

The results of this pooled analysis have recently been included in the latest consensus article on the current management of patients with desmoid tumors. Mutation status, tumor size, and tumor location could be used to screen patients at higher risk of failure of AS. This information can be used to personalize FU and potentially anticipate the need for AT. However, a period of AS should be proposed as the initial strategy for all patients affected by desmoid tumors, with very few exceptions (3).

The probability of tumor regression should be taken into account when considering initiating AT. In patients with a relatively high likelihood of tumor regression, a longer wait can be considered before addressing symptoms. Conversely, in patients with a reduced probability of regression, AT can be started earlier in case of symptoms and/or progression. Patients with DTF harboring an S45F mutation, with a larger initial tumor size, or with tumors located in the extremities or head/neck require more careful surveillance (clinical and radiological evaluation every 3–4 months), especially in the first year after diagnosis, as progression is most likely during this period (4–6). In the case of tumor progression, earlier initiation of treatment in these patients may be considered when the tumor is still smaller, potentially leading to less morbidity. As time since diagnosis increases and tumors remain stable or regress, frequent FU can be reduced (clinical and radiological evaluation every 6 months or longer in the case of regressing DTF). Patients with small tumors or abdominal wall tumors require AT less frequently and have a relatively high probability of RR development. Therefore, these patients need less frequent FU if they do not show tumor progression (clinical and radiological evaluation every 6 months or longer), and symptom-based monitoring may even be considered.

These implications cannot be extrapolated to all patients with DTF, as those with intra-abdominal tumors and familial adenomatous polyposis–related DTF were excluded from this study. Additionally, these implications must be interpreted with caution for pregnant patients because of limited data available in prospective studies. However, recent studies suggest that pregnancy does not influence DTF outcomes, and AS should also be the first-line strategy in patients with pregnancy-associated DTF (22, 23).

In conclusion, initial tumor size and tumor location should be included in the treatment algorithm for DTF, whereas *CTNNB1* mutation status may be used as additional information to tailor the management to the individual patient. The results of this study can be used to develop a more personalized approach, with more careful surveillance for patients with tumors located in the extremities and head/neck, especially when carrying an S45F mutation or other *CTNNB1* mutations compared with T41A, S45P, or wild-type mutations.

## Supplementary Material

Supplementary Figure 1 Supplementary Figure 1. CONSORT diagram of included patients.

Supplementary Figure 2 Supplementary Figure 2. Kaplan-Meier curves for treatment-free survival (A) and crude cumulative incidence curves for RECIST progression (B), RECIST regression as first event (C), regression post-RECIST progression (D), first regression (E), RECIST regression as any event (F), progression free survival (PFS) (G), on the whole series.

Supplementary Figure 3 Supplementary Figure 3. Crude cumulative incidence curves for regression post-RECIST progression according to sex (A), initial tumour size in mm (B), tumour locations (C), CTNNB1 mutation types (D)

Supplementary Figure 4 Supplementary Figure 4. Crude cumulative incidence curves for first regression according to sex (A), initial tumour size in mm (B), tumour locations (C), CTNNB1 mutation types (D).

Supplementary Figure 5 Supplementary Figure 5. Crude cumulative incidence curves for RECIST regression as any event according to sex (A), initial tumour size in mm (B), tumour locations (C), CTNNB1 mutation types (D).

Supplementary Figure 6 Supplementary Figure 6. Crude cumulative incidence curves for progression free survival as any event according to sex (A), initial tumour size in mm (B), tumour locations (C), CTNNB1 mutation types (D).

Supplementary Figure 7 Supplementary Figure 7. Flowchart of patients who received an active treatment.

Supplementary Table 1 Supplementary Table 1. Distribution of mutation and median size according to anatomical site.
