# Supplementary figures and images for: Active Surveillance in Patients with Extra-abdominal Desmoid-Type Fibromatosis: A Pooled Analysis of Three Prospective Observational Studies
Source: Clin Cancer Res. 2024 Dec 2;31(3):603–10. doi: 10.1158/1078-0432.CCR-24-2340 (PMC11788647; doi:10.1158/1078-0432.CCR-24-2340)

**Supplementary Figure 1.** CONSORT diagram of included patients.

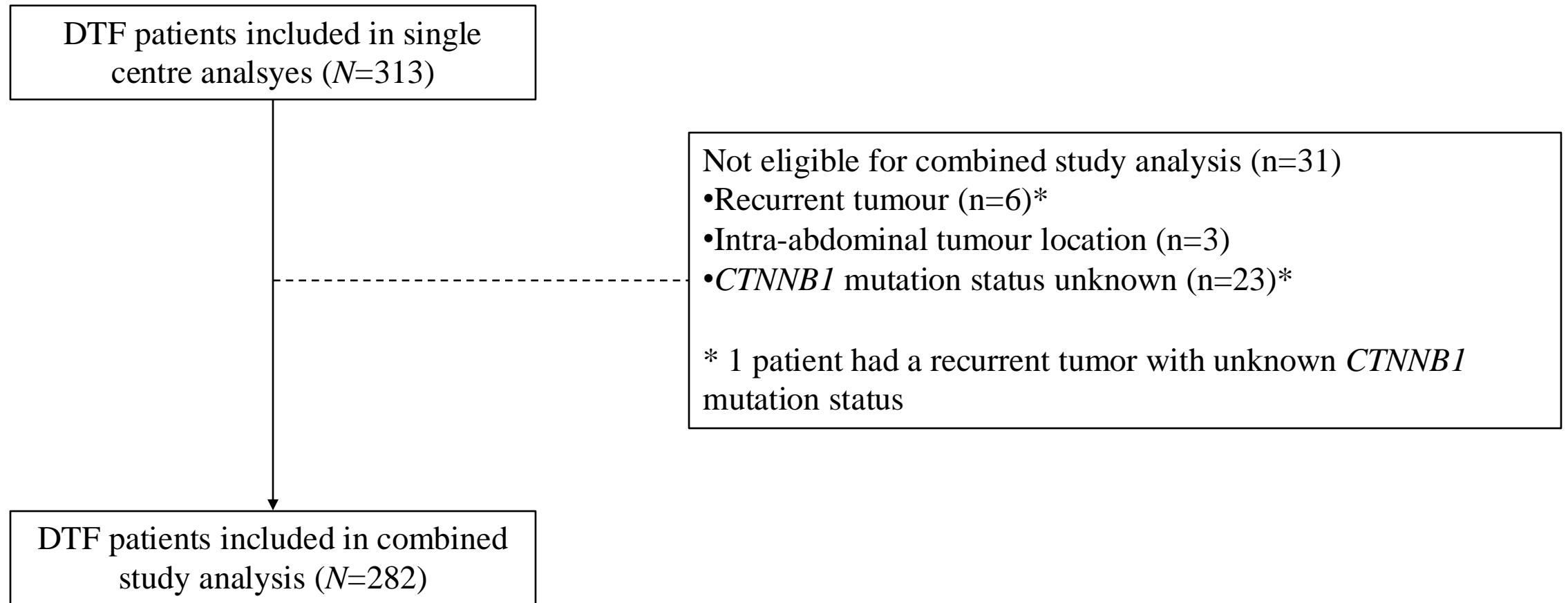

Supplement: Supplementary Figure 1 — CONSORT diagram of included patients. [file ccr-24-2340_supplementary_figure_1_suppsf1.pdf]
