# Supplementary material for: Active Surveillance in Patients with Extra-abdominal Desmoid-Type Fibromatosis: A Pooled Analysis of Three Prospective Observational Studies
Source: Clin Cancer Res. 2024 Dec 2;31(3):603–10. doi: 10.1158/1078-0432.CCR-24-2340 (PMC11788647; doi:10.1158/1078-0432.CCR-24-2340)

Supplementary Figure 2. Kaplan-Meier curves for treatment-free survival (A) and crude cumulative incidence curves for RECIST progression (B), RECIST regression as first event (C), regression post-RECIST progression (D), first regression (E), RECIST regression as any event (F), progression free survival (PFS) (G), on the whole series.

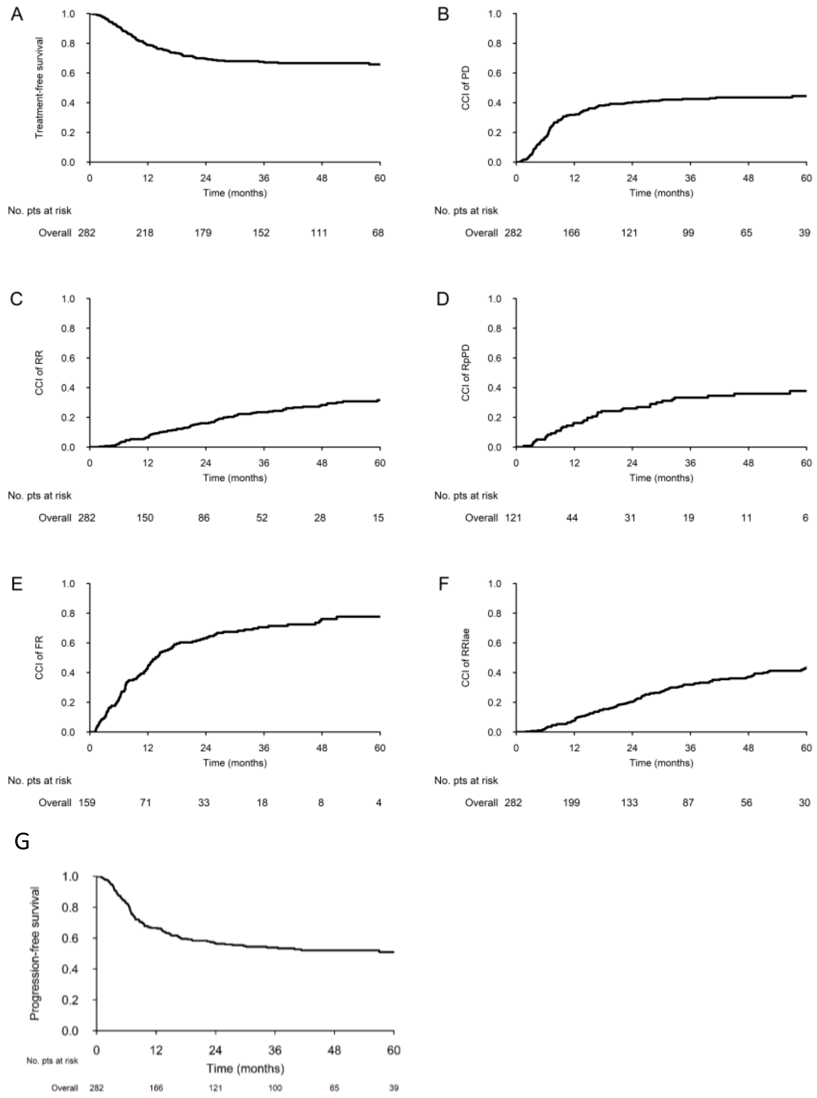

Supplement: Supplementary Figure 2 — Kaplan-Meier curves for treatment-free survival (A) and crude cumulative incidence curves for RECIST progression (B), RECIST regression as first event (C), regression post-RECIST progression (D), first regression (E), RECIST regression as any event (F), progression free survival (PFS) (G), on the whole series. [file ccr-24-2340_supplementary_figure_2_suppsf2.pdf]
