# Supplementary material for: Active Surveillance in Patients with Extra-abdominal Desmoid-Type Fibromatosis: A Pooled Analysis of Three Prospective Observational Studies
Source: Clin Cancer Res. 2024 Dec 2;31(3):603–10. doi: 10.1158/1078-0432.CCR-24-2340 (PMC11788647; doi:10.1158/1078-0432.CCR-24-2340)

Supplementary Figure 3. Crude cumulative incidence curves for regression post-RECIST progression according to sex (A), initial tumour size in mm (B), tumour locations (C), CTNNB1 mutation types (D)

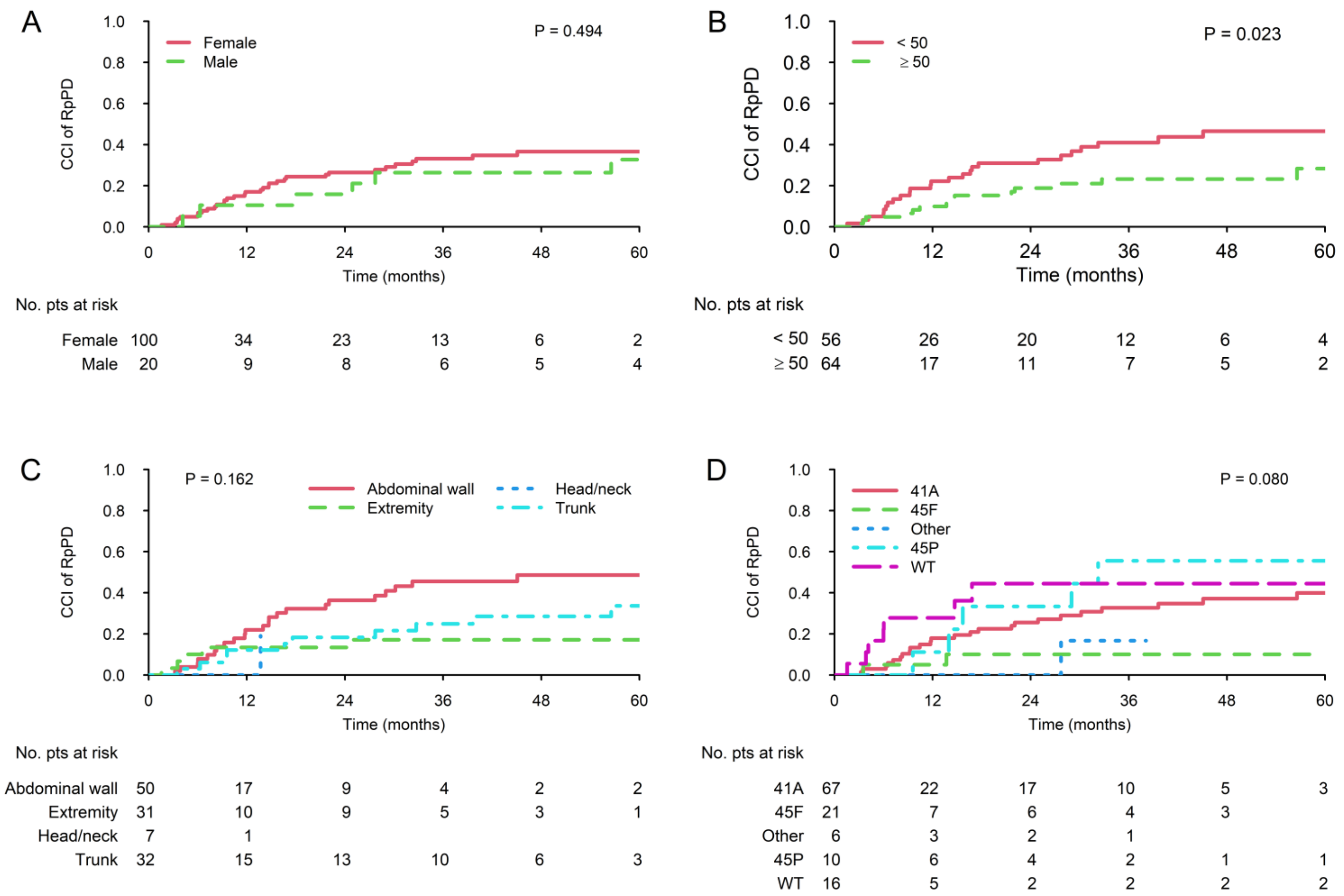

Supplement: Supplementary Figure 3 — Crude cumulative incidence curves for regression post-RECIST progression according to sex (A), initial tumour size in mm (B), tumour locations (C), CTNNB1 mutation types (D) [file ccr-24-2340_supplementary_figure_3_suppsf3.pdf]
