# Supplementary material for: Active Surveillance in Patients with Extra-abdominal Desmoid-Type Fibromatosis: A Pooled Analysis of Three Prospective Observational Studies
Source: Clin Cancer Res. 2024 Dec 2;31(3):603–10. doi: 10.1158/1078-0432.CCR-24-2340 (PMC11788647; doi:10.1158/1078-0432.CCR-24-2340)

Supplementary Figure 5. Crude cumulative incidence curves for RECIST regression as any event according to sex (A), initial tumour size in mm (B), tumour locations (C), CTNNB1 mutation types (D).

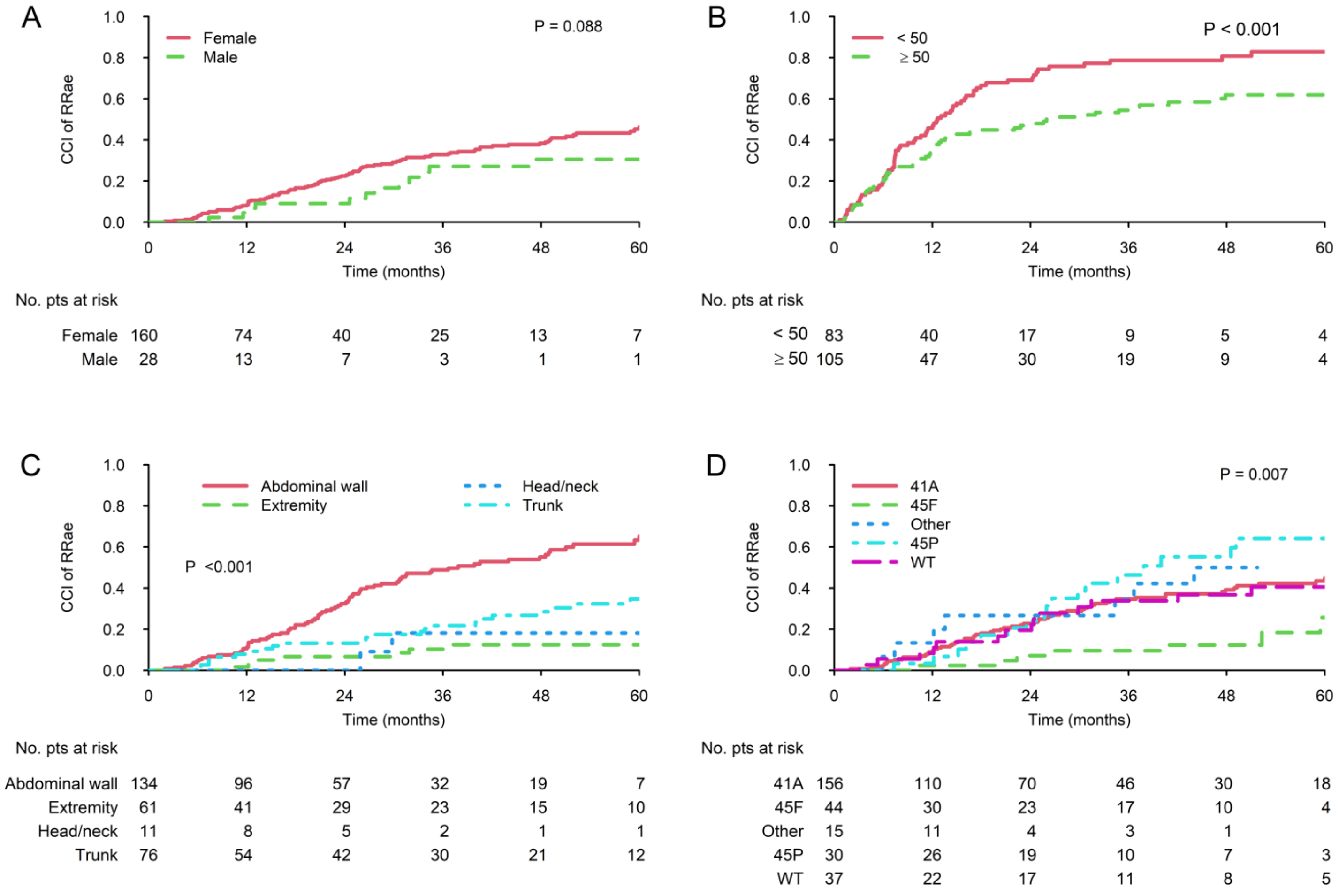

Supplement: Supplementary Figure 5 — Crude cumulative incidence curves for RECIST regression as any event according to sex (A), initial tumour size in mm (B), tumour locations (C), CTNNB1 mutation types (D). [file ccr-24-2340_supplementary_figure_5_suppsf5.pdf]
