# Supplementary material for: Active Surveillance in Patients with Extra-abdominal Desmoid-Type Fibromatosis: A Pooled Analysis of Three Prospective Observational Studies
Source: Clin Cancer Res. 2024 Dec 2;31(3):603–10. doi: 10.1158/1078-0432.CCR-24-2340 (PMC11788647; doi:10.1158/1078-0432.CCR-24-2340)
Supplement: Supplementary Figure 7 — Flowchart of patients who received an active treatment. [file ccr-24-2340_supplementary_figure_7_suppsf7.pdf]

Supplementary Figure 7. Flowchart of patients who received an active treatment

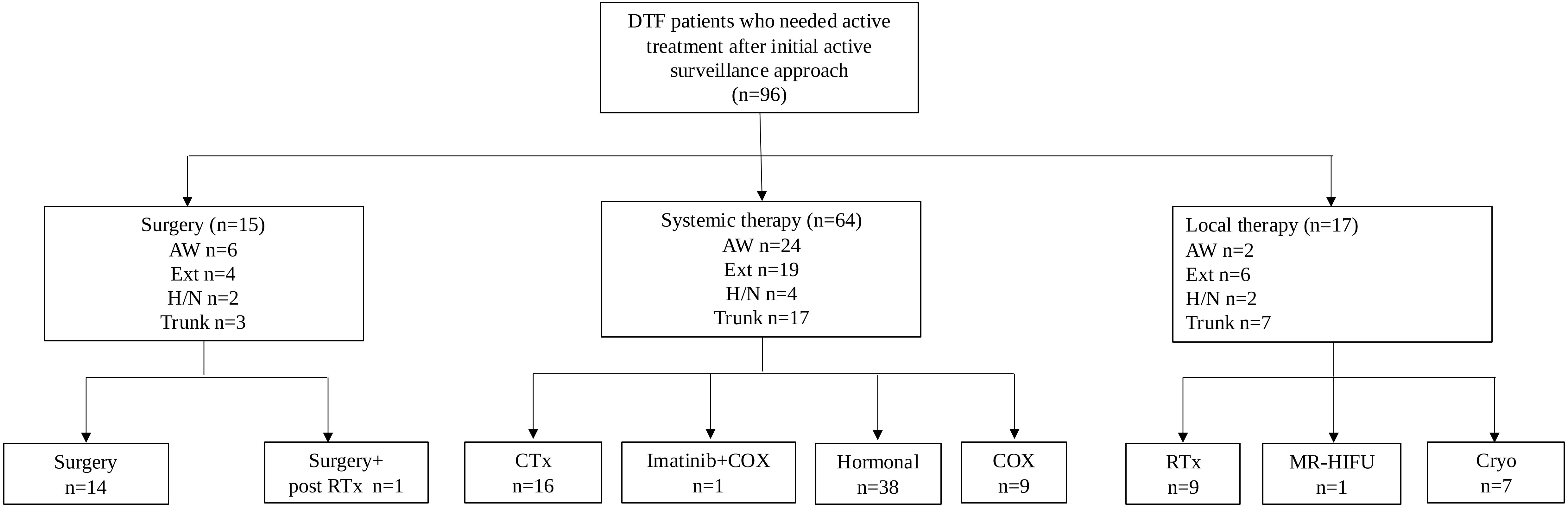

Abbreviations: AW, abdominal wall; Ext, extremity; H/N, head/neck; post-RTx, post-operative radiotherapy; CTx, chemotherapy; COX, Cox inhibitors; Hormonal, hormonal therapy; RTx, radiotherapy; MR-HIFU, magnetic resonance high intensity focused ultrasound; Cryo, cryotherapy
