# Supplementary material for: Active Surveillance in Patients with Extra-abdominal Desmoid-Type Fibromatosis: A Pooled Analysis of Three Prospective Observational Studies
Source: Clin Cancer Res. 2024 Dec 2;31(3):603–10. doi: 10.1158/1078-0432.CCR-24-2340 (PMC11788647; doi:10.1158/1078-0432.CCR-24-2340)
Supplement: Supplementary Table 1 — Distribution of mutation and median size according to anatomical site. [file ccr-24-2340_supplementary_table_1_suppst1.docx]

| Supp Table 1. Distribution of mutation and median size according to anatomical site | | | | |
| --- | --- | --- | --- | --- |
|  | Abdominal wall (134) | Head&Neck (11) | Extremities (61) | Trunk (76) |
| T41A (*n*=156) | 75 (56%) | 5 (45%) | 31 (51%) | 45 (60%) |
| S45F (*n*=44) | 10 (8%) | 3 (27%) | 16 (26%) | 15 (19%) |
| S45P (*n*=30) | 20 (15%) | 1 (9%) | 3 (5%) | 6 (8%) |
| Other (*n*=15) | 11 (8%) | 0 | 0 | 4 (5%) |
| WT (*n*=37) | 18 (13%) | 2 (19%) | 11 (18%) | 6 (8%) |
| Median size (cm) | 46 | 55 | 64 | 50 |
